# Triglyceride to high-density lipoprotein cholesterol and low-density lipoprotein cholestrol to high-density lipoprotein cholesterol ratios are predictors of cardiovascular risk in Iranian adults: Evidence from a population-based cross-sectional study

**DOI:** 10.22088/cjim.11.1.53

**Published:** 2020

**Authors:** Karimollah Hajian-Tilaki, Behzad Heidari, Afsaneh Bakhtiari

**Affiliations:** 1 Social Determinants of Health Research Center, Health Research Institute, Babol University of Medical Sciences, Babol, Iran; 2 Mobility Impairment Research Center, Health Research Institute, Babol University of Medical Sciences, Babol, Iran; 3 Department of Midwifery, Babol University of Medical Sciences, Babol, Iran

**Keywords:** Triglyceride, HDL- cholesterol, Triglyceride/HDL-cholesterol ratio, LDL-cholesterol, LDL-cholesterol/HDL-cholesterol ratio, Cardiovascular risk

## Abstract

**Background:**

The superiority of TG/HDL-C and LDL-C/HDL-C ratios in predicting CVD risk is a matter of debates. Thus, the objective of this study was to compare TG/HDL-C and LDL-C to HDL-C ratios in predicting the risk of CVD events.

**Methods:**

In a population-based cross-sectional study, 567 representative participants aged 40 years or older were entered in the study in Babol, North of Iran. The demographic data, anthropometric measures, and the cardio metabolic risk factors were measured. The individual risk of CVD events was assessed by ACC/AHA risk model. ROC analysis was applied to estimate the diagnostic accuracy and the optimal cut-off points of TG/HDL-C and LDL-C/HDL-C ratios.

**Results:**

The AUC of TG/HDL-C and LDL-C/HDL-C ratios were rather similar and both parameters significantly predicted CVD risk in men comparably, and TG/HDL-C at optimal cutoff point of 3.6 produced 75% sensitivity and 39% specificity. However,in women TG/HDL-C with AUC of 0.65( p=0.091) at optimal cutoff value of 3.4 produced a sensitivity of 82% and specificity of 51%. The LDL-C/HDL-C ratio had no discriminative ability in predicting CVD risk in women.

The adjusted OR of TG/HDL-C at 2nd quartile was significant (OR=3.22, 95% CI:1.25-8.29) and a greater association was found with 3rd and 4rth quartiles

**Conclusion:**

Both TG/HDL–C and LDL-C/HDL-C ratios comparably predict CVD risk in men, whereas in women only TG/ HDL-C is a significant predictor but not LDL-C/HDL-C.

Cardiovascular diseases as a major public health problem worldwide are the main causes of death both in the developing and industrial countries (1). Metabolic syndrome (MetS) and diabetes alone and/or in combination are also predictors of cardiovascular mortality (2). It is well-established that obesity, insulin resistance and hyperlipidaemia are the central components of MetS (2, 3). There is evidence that the pattern of cardiovascular risk factors and their contribution in mortality have changed dramatically in Iranian population in recent decades (4, 5). According to the epidemiological transition model, the Iranian population had experienced cardiovascular events rapidly because of changing toward western lifestyles. 

A high prevalence of obesity, dyslipidemia, impaired glucose tolerance, type 2 diabetes, and metabolic syndrome were responsible for the development of higher than expected mortalities in Iranian populations (6-10). The high level of triglyceride (TG) and the low level of high-density lipoprotein cholesterol (HDL-C) are associated with insulin resistance, type 2 diabetes and thus metabolic syndrome (11-13). Both conditions have been considered as components of metabolic syndrome by the World Health Organization (WHO), International Diabetes Federation (IDF), Adult Treatment Panel (ATP III) and National Heart Lung and Blood Institute/American Heart Association (NHLBI/AHA) (13-16). 

Additionally, a single dimensional measure has been defined by TG/ HDL-C ratio, as an index of heart disease mortality and incidence of type 2 diabetes mellitus in men (17). As a TG/HDL ratio of > 3.5 is associated with impaired glucose tolerance, type 2 diabetes, atherogenic dyslipidaemia and metabolic syndrome (17), and particularly in obese and diabetic patients as well as in obese children. This measure is associated with an increased risk of cardiovascular disease and its consequent mortality (19-21). Furthermore, low-density lipoprotein cholesterol (LDL-C) plays a basic metabolic role in the pathogens of cardiovascular disease (CVD) (22, 23), but its independent association with MetS has not been established yet clearly, so it was not considered as a component of MetS (24). On the other hand, small dense low-density lipoprotein (sd-LDL) particles are also linked with CVD risk (25, 26) but their application as a routine biochemical test is not clinically applicable. Nevertheless, the ratio of low density cholesterol to high-density lipoprotein cholesterol (LDL-C/HDL-C ratio) provides a simple measure to identify dyslipidaemic patients. 

The present evidence suggested that the LDL-C /HDL ratio was an independent predictor of coronary stenosis and coronary artery plaques (27). However, the optimal cutoff values of both LDL-C/HDL-C and TG/HDL-C as one-dimensional ratio indexes are dependent on ethnicity and gender (28). Therefore, the superiority of these two ratio measures and their optimal cutoff values in predicting cardiovascular risk in the general population remain to be determined. Since the relative ability of TG- to- HDL-C ratio and LDL-C –to- HDL-C ratios in recognizing the cardiovascular risk in general population have not been established yet, data in this regard in Iranian adult population are also scarce. We, therefore, performed the present study to examine and compare the ability of these measures in predicting 10-year risk of CVD by using the American College of Cardiology/American Heart Association (ACC/AHA) risk model (29).

## Methods

The data of this study extracted from a population-based cross-sectional of Babol Glucose and Lipid study was originally designed to examine the prevalence of cardiometabolic risk factors in a population living in a geographic region located in the south of the Caspian Sea, North of Iran in 2012. The original data included one thousand representative samples of the adult population aged 20 to 70 years. The two-stage cluster sampling method was used in the selection of the study subjects in the urban community. All participants had given a written consent prior to participation in the study and the study protocol was approved by the Ethics Committee of Babol University of Medical Sciences. For the current analysis, 567 subjects without a prior history of CVD events aged 40 years or older entered the study. 

**Data collection: **Details of sample selection and data collection were described elsewhere (8). In brief, 25 random clusters were selected at community under coverage of health care systems and within each cluster, about 40 subjects were selected. All participants were interviewed and clinically examined at home visit in a family health survey. The data regarding demographic characteristics, anthropometric measures and blood pressure (systolic and diastolic blood pressure (SBP), diastolic blood pressure (DBP) were provided at home visit and from them, the mean arterial pressure(MAP) was calculated as MAP=DBP+0.33 (SBP-DBP).Then all participants were invited to the central lab of the Ayatollah Rouhani Hospital with 10-12 hours overnight fasting for assessment of total cholesterol (TC), TG, HDL-C and, LDL-C and fasting blood sugar (FBS) with the enzymatic method. The ratios of TG to HDL-C and LDL-C to HDL-C were calculated for all participants. The body mass index was calculated as weight in kg dividing by height in m^2^ and the waist circumference >102 cm for men and >88 cm for women were considered as abdominally obese.

**Statistical Analysis: **In statistical analysis, we used SPSS software Version 18 and Stata software Version 13.0. First, ACC/AHA 10-year risk model of CVD events was applied to assess the individual risk of CVD based on cardiometabolic risk profiles of the participants (24). The sex-ethnic baseline risk and the related coefficients of the Cox regression model suggested by ACC/AHA were used for individual risk assessment. We used the Caucasian ethnic baseline risk that corresponds to the ethnicity of the study population. Then, the quantitative risk was dichotomized into two categories as low risk (<10%) and high risk (≥10%). In bivariate analysis, the low and high-risk groups were compared with regard to TG/HDL-C, LDL-C /HDL-C ratios, and other cardiometabolic risk factors using independent sample t-test for normally distributed data and Wilcoxon rank test for variables with non-normal distribution according to gender. ROC curve analysis was applied to estimate the accuracy of TG/HDL-C and LDL-C/HDL-C ratios in differentiating high risk versus low-risk individuals with the calculation of the area under the curve (AUC) obtained from the same study population. The covariance structure of Delong method with Stata software was used to characterize and test the superiority of either of these two ratio biomarkers. The optimal cutoff value of each biomarker was estimated using Youdens' index that maximizes the total correct classification which was determined by sensitivity+specificity-1. This index maximizes sensitivity and specificity. Then the sensitivity and specificity of each measure were determined at the optimal cutoff point. The diagnostic accuracy of each ratio index as defined by the area under ROC curve (AUC) was determined with 95% confidence interval. The ability of higher tops quartiles versus 1st quartile of TG/HDL-C and LDL-C /HDL-C ratios in association with high risk versus low-risk individuals was determined with calculation of odds ratio (OR) and the corresponding 95% confidence interval using logistic regression model after adjustment for age, sex and educational level, BMI, abdominal obesity, mean arterial pressure, and FBS. In all analysis, we used two-sided test and the p-values less than 0.05 were considered as significant level.

## Results

The mean age of the male and female participants were 53.4±8.9 and 51.3±8.2 years respectively. The mean value of TG/HDL-C ratio was significantly greater in men than women (5.79±4.22 versus 3.67±1.32, P=0.04), whereas, the mean value for LDL-C /HDL-C ratio did not differ between the two sexes (3.54±1.31 versus 3.67±1.32, P=0.25). Overall, 28% of the participants were in high (≥10%) CVD risk group (42.5% men versus 15.1% women, P=0.001). In table 1, men and women in high and low CVD risk groups were compared according to cardiometabolic risk factors as well as TG/HDL-C and LDL-C/HDL-C ratios. In men, all metabolic risk factors, in particular, TG/ HDL-C and LDL-C/HDL-C ratios were significantly higher in high risk compared to low-risk group, but in women, the pattern was rather different and a significant difference was observed only between TG, FBS, SBP, DBP, and TG/HDL ratio, whereas, the difference in LDL-C, HDL-C, BMI, WC, and LDL-C/HDL-C ratio did not reach to a statistically significant level.

**Figure 1:**
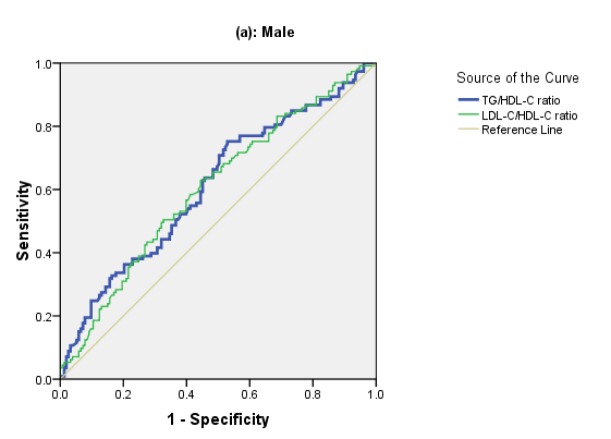
ROC curve of TG/HDL-C and LDL-C/HDL-C ratios in predicting CVD risk, panel (a): Male, panel (b): Female

Table 2 summarizes the diagnostic accuracy of TG/HDL-C and LDL-C/HDL-C ratio and their optimal cutoff values in predicting CVD events according to sex. In men, the TG/HDL-C and LDL-C/HDL-C ratios yielded similar accuracy in differentiating low and high-risk groups at a significant level, and the TG/HDL-C ratio at cutoff level of 3.6 yielded a sensitivity of 75% and specificity of 39%. However, the predictive accuracy (AUC) of TG/HDL-C ratio in women was greater than men (P=0.001), and at cutoff value of 3.4 yielded a sensitivity and specificity of 82% and 41%, respectively. While LDL-C/HDL-C ratio in women showed no discriminative ability in differentiating high versus low CVD risk group (AUC=0.53, P=0.46). Figure 1 shows the ROC curves for the two ratio biomarkers in panel (a) for men and panel (b) for women.

In table 3, the association of higher quartile versus 1st quartile for both biomarkers with cardiovascular risk was shown by unadjusted OR after controlling for age, sex, educational level, BMI, abdominal obesity, mean arterial pressure, and FBS. The strength of association increased further at higher quartiles after adjustment. The ability of TG/HDL-C in the differentiation of high from low-risk CVD risk group at 4th quartile was 4.51 (95% CI: 1.80–11.25, P= 0.001) times greater than the 1st quartile. The association of LDL-C/HDL-C ratio reached to a statistically significant at 3rd and 4th quartiles after adjustment, but not at 2nd quartile level. Further stratified analysis by gender showed no significant association between quartiles of LDL-C/HDL- ratio and CVD risk in women but the magnitude of adjusted OR for TG/HDL-C was inefficiently large in women since, the data of outcome of interest (>10% CVD risk) was sparse in the 1st quartile of TG/HDL-C in women.

**Table 1:** The mean (SD) of cardiometabolic risk factors in low and high cardiovascular risk according to sex

**Cardiometabolic risk factors**	**CVD risk**		**P-value**
	**<10%** **Mean (SD)**	**>=10%** **Mean (SD)**	
**Men** Cholesterol (mg/dl)	48.1 (5.6)	60.5 (7.5)	0.001
TG (mg/dl)	190.6 (38.9)	210.4 (78.5)	0.007
LDL-C (mg/dl)	180.8 (115.3)	215.8 (134.4)	0.02
HDL-C (mg/dl)	118.7 (36.3)	127.4 (43.9)	0.08
FBS (mg/dl)	105.0 (32.5)	128.5 (48.8)	0.003
BMI (kg/m^2^)	26.3 (3.6)	27.6 (6.8)	0.03
WC (cm)	93.0 (13.3)	96.9 (15.9)	0.04
DBP (mm/Hg)	81.9 (12.9)	87.3 (13.4)	0.001
SBP (mm/Hg)	126.1 (13.9)	135.5 (17.3)	0.001
TG/HDL-C	5.14 (3.74)	6.69 (4.68)	0.003
LDL-C/HDL-C	3.33 (1.24)	3.81 (1.35)	0.003
**Women**			
Cholesterol(mg/dl)	211.6 (42.1)	222.1 (51.6)	0.13
TG(mg/dl)	171.4 (101.2)	241.3 (223.4)	0.001
LDL-C(mg/dl)	137.6 (40.9)	137.7 (47.0)	0.98
HDL-C(mg/dl)	40.5 (15.7)	36.7 (8.6)	0.12
FBS(mg/dl)	11.6 (40.7)	168.7 (88.3)	0.001
BMI(kg/m^2^)	29.9 (5.7)	30.9 (5.7)	0.25
WC(cm)	95.5 (15.4)	99.1 (15.3)	0.16
DBP(mm/Hg)	84.2 (15.6)	91.7 (17.5)	0.004
SBP(mm/Hg)	127.6 (18.3)	149.4 (18.3)	0.001
TG/HDL-C	4.67 (3.46)	7.13 (7.87)	0.001
LDL-C/HDL-C	3.63 (1.29)	3.88 (1.49)	0.25

CVD = Cardiovascular disease; TG=Triglycerides; LDL= Low density lipoprotein; HDL= High density lipoprotein cholesterol; FBS= Fasting blood sugar

BMI = Body mass index; WC =Waist circumference; DBP= Diastolic blood pressure; SBP= Systolic blood pressure

**Table 2:** The diagnostic accuracy of TG to HDL-C ratio and LDL-C to HDL-C ratio in predicting cardiovascular risk and their optimal cut-off values

**Predictors**	**AUC (95% CI)**	**P-value**	**Optimal** **cut-off**	**Sensitivity**	**Specificity**
**Men**					
TG/HDL-C	0.61 (0.54, 0.68)	0.002	3.6	0.75	0.39
LDL-C/HDL-C	0.60 (0.53, 0.67)	0.004	3.2	0.68	0.42
**Women**					
TG/HDL-C	0.65 (0.57, 0.73)	0.001	3.4	0.82	0.41
LDL-C/HDL-C	0.53 (0.44, 0.63)	0.46	3.1	0.69	0.47
**All **					
TG/HDL-C	0.63 (0.58, 0.68)	0.001	2.7	0.89	0.24
LDL-C/HDL-C	0.56 (0.51, 0.61)	0.027	2.7	0.85	0.21

AUC= Area under the roc curve; TG=Triglycerides; LDL= Low density lipoprotein; HDL= High density lipoprotein cholesterol

TG=Triglycerides; LDL-= Low density lipoprotein; HDL= High density lipoprotein cholesterol; CVD = cardiovascular disease

**Table 3:** The unadjusted and adjusted odds ratio (OR) of TG/HDL-C and LDL-C/HDL-C ratios and the 95% confidence interval (CI) in predicting cardiovascular risk

**Cardio metabolic risk factors**	**Unadjusted OR** **(95% CI)**	**P-value**	**Adjusted** ^╞^ **OR** **(95% CI)**	**P-value**
TG/HDL-C		0.001		0.012
1^st^ quartile	1 ( ref)	-	1 (ref)	-
2^nd^ quartile	2.51 (1.37, 4.60)	0.003	3.22 (1.25, 8.39)	0.02
3^rd^ quartile	2.59 (1.41, 4.74)	0.002	3.85 (1.48, 9.99)	0.005
4rth quartile	4.28 (2.38, 7.72)	0.001	4.51 (1.80, 11.25)	0.001
LDL-C/HDL-C		0.22		0.001
1^st^ quartile	1 (ref)	-	1 (ref)	-
2^nd^ quartile	1.10 (0.64, 1.90)	0.78	1.06 (0.44, 2.50)	0.89
3^rd^ quartile	1.47 (0.87, 2.48)	0.15	3.18 (1.38, 7.33)	0.007
4rth quartile	1.62 (0.95, 2.73)	0.07	4.46 (1.91, 10.42	0.001

The odds ratio was adjusted by age, sex, educational level, body mass index (BMI), abdominal obesity,

mean arterial pressure (MAP) and fasting blood sugar (FBS).

## Discussion

These findings indicated that 28% of the study population especially male subjects were at high risk for developing cardiovascular events, and cardiometabolic risk factors were more prevalent in high CVD risk group compared to low-risk group. In this setting the TG/ HDL-C ratio at cut-off level of 3.6 recognized high CVD risk group at sensitivity of 75% and specificity of 39% and LDL-C/HDL-C ratio with cut-off point of 3.5 yielded a sensitivity and specificity of 82% and 41%, respectively for differentiation of high and low CVD risk groups. The association of TG/HDL-C with CVD risk at higher quartiles was stronger. Both indexes yielded similar accuracy in men, but in women, LDH-C/HDL-C ratio was not associated with CVD risk.

These findings are in agreement with the results of earlier studies which have addressed the association between TG/HDL-C ratio and risk factors of CVD events such as diabetes, metabolic syndrome, dyslipidemia, hypertension, and insulin resistance (19, 25, 30-34). The association between TG/HDL-C and cardiovascular events has also been shown in two longitudinal studies of Iranian populations (28, 31). In another longitudinal study of 39447 men, the predictive ability of TG/HDL-C ratio was comparable or better than the metabolic syndrome. In this study, TG/HDL-C ratio > 3.5 predicted future development of cardiovascular diseases and coronary heart deaths, and the annual incidence of diabetes was 2 times higher as compared with TG/HDL-C ratio < 3.5 (17). In the Korean National Health and Nutrition Examination Survey in 2013 and 2014, the TG/HDL-C ratio at cutoff point ≥ 3.52 was significantly associated with metabolic syndrome (33). The TG/HDL-C ratio was also associated with hypertension, hypercholesterolemia, and hypertriglyceridemia in both sexes (30). In overweight and obese, hypertensive individuals, higher TG/HDL-C ratio is a predictor of cardiovascular diseases and a higher incidence of fatal and nonfatal cardiovascular events (19, 35). In conditions with an inflammatory process such as patients on haemodialysis and rheumatoid arthritis (36, 37), the elevated TG/HDL-C ratio is associated with cardiovascular deaths and overall-cause death (38). Even in healthy adults and children, high TG/HDL-C ratio is a marker for future development of cardiovascular diseases (21, 39). 

The results regarding the ability of high LDL-C/HDL-C ratio in recognizing high-risk individuals for CVD events in men, as found in this study, are also in line with other studies (27, 40, 42). One large study of subjects aged > 35 years old found that LDH-C/HDL-C ratio was a marker of hypertension and hypercholesterolemia in both men and women in a Canadian population (40). In another study of 101 Japanese, the LDL-C -to-HDL-C ratio was an independent predictor of coronary artery stenosis and vulnerable coronary artery plaque in diabetic patients (27). In contrast, in a prospective study of 356 patients with acute intracranial hemorrhage, patients at the lowest quartiles of LDL-C /HDL-C ratio had the highest cumulative incidence rates of all-cause mortality with 3.55- fold increase in the risk of all-cause mortality as compared with highest quartiles (41). Based on available data, elevated serum triglycerides is commonly associated with reduced HDL-C and increased number of small dense low-density lipoprotein (17). Triglycerides themselves are strong risk factors for cardiovascular events but stratifying by HDL-C levels provides a more accurate measure in detecting high-risk individuals (43). Nevertheless, an analysis of data from the cohort of the Quebec Cardiovascular Study during a 13-year follow-up period, revealed a significant association between the Log TG/HDL ratio and features of LDL size phenotype which was comparable to TG alone (44). Although, increased levels of some lipids are associated with higher risk of coronary heart disease, but, cardiac risk of cardiovascular disease for small dense LDL particles has been established. However, routine assessment of these lipoproteins is not practical instead, several indexes including TG/LDL-C ratio, the ratio of total cholesterol to HDL-C and, to a lesser extent, the ratio of LDL-C to HDL have been used to assess atherogenic lipoproteins status for predicting coronary artery disease. Among several indexes, ratios that have atherogenic particles in the numerator and HDL-C or its constituents in the denominator yield stronger ability in predicting cardiovascular disease. As a result, TG/HDL-C ratio proved to be a highly significant independent predictor of myocardial infarction, even stronger than TC/HDL-C and LDL-C /HDL-C, because, triglycerides have high plasma concentration of lipoprotein for generation of small dense LDL (31, 43). In addition, the TG/HDL-C ratio is a marker of insulin resistance which acts differently regarding ethnicity and sex and correlates with other atherogenic lipids such as TG-rich lipoproteins, remnant, and small dense LDL particles. Moreover, it can be used as a measure of glycemic control especially in obese patients with type 2 diabetes (20). As shown in this study, the predictive ability of TG/HDL-C has been addressed in other Iranian populations (28, 31). One large prospective study of Iranian men aged > 40 years old, free of cardiovascular diseases, over 6.5 years of follow-up period cardiovascular events occurred in 15.5% of subjects in the top quartile and 4.9% in the first quartile. The prevalence of metabolic syndrome in subjects with TG/HDL ratio > 6.9 reached 63.6% versus 3% in those with TG/HDL ratio < 2.8 (31). Based on the findings of the present study, 75% of high-risk CVD events in men and 0.82% of women can be predicted by TG/HDL-C ratio > 3.6, and >3.4, respectively. However, the specificity of both TG/HDL-C and LDL/HDL ratios was low, so about 60% of subjects of the low-risk group for CVD may yield false positive results. Nonetheless, the identification of high-risk individuals by using an inexpensive measure is of particular importance. Especially, the ability of these measures in recognizing apparently healthy young adult and children who are at risk of future development of cardiovascular events provides an opportunity for preventive measures.

This study has limitations regarding study design which is cross-sectional and the association does not indicate causality. In addition, in calculating risk in ACC/AHA model, the baseline risk was taken into account from non-Hispanic Caucasian. This ethnic group might have rather a similar baseline risk to Iranian population according to similar ethnicity. 

 Although the confounding effects of several associated factors of CVD, such as age, sex, BMI, abdominal obesity, diabetes, hypertension and thus components of metabolic syndrome (except for lipid profiles) have been controlled by logistic regression model, the effects of unknown covariates could not be ignored. However, the results are probably less confounded since the distributions of unknown covariates are expected to be similar across comparison groups. However, the population-based subject selection, performing a standard sampling procedure with adequate sample size and appropriate method of data collection and analysis enhance the validity and reliability of the study.

In conclusion, this study indicates that TG /HDL-C and LDL-C /HDL-C ratios are easy and accessible measures in recognizing individuals who are at higher risk of CVD. However, this issue requires a longitudinal study of healthy individuals, and serial measurement of TG, LDL-C, HDL-C and other associated factors of cardiovascular disease. Meanwhile, compareson of individuals with and without high TG/HDL-C and LDL-C/HDL-C ratios over the follow-up period can be of help in the demonstration of their predictive abilities.
